# Large-scale variation in density of an aquatic ecosystem indicator species

**DOI:** 10.1038/s41598-018-26847-x

**Published:** 2018-06-12

**Authors:** Chris Sutherland, Angela K. Fuller, J. Andrew Royle, Matthew P. Hare, Sean Madden

**Affiliations:** 1 Department of Environmental Conservation, University of Massachusetts, Amherst, 01003 USA; 2 Department of Natural Resources, U.S. Geological Survey, New York Cooperative Fish and Wildlife Research Unit, Cornell University, Ithaca, 14853 USA; 3 U.S. Geological Survey, Patuxent Wildlife Research Center, Laurel, 12311 USA; 4 Department of Natural Resources, Cornell University, Ithaca, 14853 USA; 5 New York State Department of Environmental Conservation, Division of Fish and Wildlife, Albany, 12233 USA; 6 U.S. Geological Survey, New York Cooperative Fish and Wildlife Research Unit, Department of Natural Resources, Cornell University, Ithaca, 14853 USA

**Keywords:** Population dynamics, Population dynamics, Environmental impact

## Abstract

Monitoring indicator species is a pragmatic approach to natural resource assessments, especially when the link between the indicator species and ecosystem state is well justified. However, conducting ecosystem assessments over representative spatial scales that are insensitive to local heterogeneity is challenging. We examine the link between polychlorinated biphenyl (PCB) contamination and population density of an aquatic habitat specialist over a large spatial scale using non-invasive genetic spatial capture-recapture. Using American mink (Neovison vison), a predatory mammal and an indicator of aquatic ecosystems, we compared estimates of density in two major river systems, one with extremely high levels of PCB contamination (Hudson River), and a hydrologically independent river with lower PCB levels (Mohawk River). Our work supports the hypothesis that mink densities are substantially (1.64–1.67 times) lower in the contaminated river system. We demonstrate the value of coupling the indicator species concept with well-conceived and spatially representative monitoring protocols. PCBs have demonstrable detrimental effects on aquatic ecosystems, including mink, and these effects are likely to be profound and long-lasting, manifesting as population-level impacts. Through integrating non-invasive data collection, genetic analysis, and spatial capture-recapture methods, we present a monitoring framework for generating robust density estimates across large spatial scales.

## Introduction

Measuring ecosystem responses to variation in the environment is challenging and requires geographically distributed research networks^1^ or the prioritization of focus towards ecologically important ‘surrogate’ species within the ecosystem^2^. While there are challenges associated with the surrogate species concept^3,4^, it offers a pragmatic approach that is widely applied, particularly in the case of environmental risk^5^. In general, however, the minimum requirement of an indicator is that there is documented and measurable evidence of sensitivity to the perturbation in question^6^. Here, we investigate spatial variation in American mink (*Neovison vison*) density in two river systems in New York, USA, that differ in their level of polychlorinated biphenyl (PCB) contamination.

The U.S. Environmental Protection Agency (EPA) has estimated that the two General Electric Company (GE) manufacturing facilities located in Fort Edward and Hudson Falls, New York, discharged up to 1.3 million pounds of PCBs into the river^7^, but the actual amount of PCBs discharged to the river, while unknown, could be significantly higher. These unprecedented levels of discharge, and the subsequent releases from fractured bedrock, and erosion of contaminated soils and sediments have contaminated the riparian habitats including the river water, sediments, flood plains, and biota^8^, with the potential to influence the ecology of fish and wildlife that inhabit these systems. A natural resource damage assessment (NRDA) is being conducted to determine compensation for natural resource injuries from PCB contamination of the Hudson River by General Electric^9^.

American mink are an ideal indicator species for aquatic ecosystems: they are relatively short lived (≤4 years) semi-aquatic generalist predators that inhabit riparian, lake shore, wetland, and coastal habitats^10^ and are widespread in the Hudson River Valley. Their association with aquatic habitats means mink are particularly vulnerable to PCB exposure through water, sediment and soils, and through their diet of fish, aquatic invertebrates and small mammals^11,12^. In addition to exposure, they are also known to be sensitive to PCB contamination. For example, PCB contamination has been linked to jaw lesions^13,14^ and reproductive impairments such as reduced kit growth and increased kit mortality^15–17^. In the Hudson River Valley, PCB burdens found in mink are positively associated with local environmental contamination levels^18,19^ and in known contaminated areas, these burdens were 2.8 times the levels responsible for reproductive impairment^20^, and 6.6 times the levels that can lead to potential health impairment^21^, whereas in uncontaminated or less contaminated areas, burdens were generally below the levels that induce adverse toxicological effects^21^.

As part of a natural resource damage assessment, our objective was to quantify differences in mink abundance between the contaminated Hudson River system and a neighboring river system with natural contamination levels. Individual variation in vital rates has the potential to influence population dynamics^22^, and given the PCB exposure history of mink in the contaminated parts of the system, detrimental effects (e.g., reduced survival and reproduction) can manifest as observable relative reductions in occupancy and/or abundance^23^. Recently reported differences in abundance^18^ and occurrence^24^ that have been attributed to PCB levels are limited by small sample sizes and potential negative bias in both state variables through a failure to account for imperfect detection^25,26^. In order to directly compare mink density, we used spatial capture-recapture methods (SCR^27,28^) to generate spatially explicit detection-corrected estimates of mink density across large spatial scales that allow comparisons to be made between the two river systems.

Here we provide spatially explicit landscape level estimates of mink density for the Hudson River drainage where elevated levels of PCBs have been reported, and compare them to estimates of mink density in a reference river drainage, the Mohawk River. To achieve these objectives we used a combination of well-established ecological monitoring and analysis techniques: non-invasive collection of mink scat found using dogs specialized in mink-scat detection, individual identification of all collected mink scats using multilocus genotypes, and estimating density using spatial capture-recapture models applied to spatial encounter histories of uniquely identified individuals.

## Results

In 2013, 138.10 *km* and 152.64 *km* of waterway were surveyed in the Hudson River and Mohawk River, respectively. In 2014, 540.22 *km* and 571.23 *km* of waterway were surveyed in the Hudson River and Mohawk River, respectively (Table 1). Of the 108 individuals detected in the Hudson, 44 were female (9 in 2013 and 35 in 2014), 39 were male (13 in 2013 and 26 in 2014) and we were unable to determine sex for 25 individuals (8 in 2013 and 17 in 2014). In 2013, only 9 individuals were observed at >1 trap, 3 of which were observed at more than one transect, whereas in 2014, 38 were observed at >1 trap, 21 of which were observed at different transects. Of the 208 individuals detected in the Mohawk, 85 were female (29 in 2013 and 56 in 2014), 70 were male (34 in 2013 and 36 in 2014) and 53 individuals had undetermined sex (22 in 2013 and 31 in 2014). In 2013, 34 individuals were observed at >1 trap, 18 of which were observed at more than one transect, whereas in 2014, 63 were observed at >1 trap and 20 were observed at multiple transects (Supplementary Table S1). Transect and scat summaries are provided in Table 1.

**Table 1:** Summaries of the number of sites visited, scat samples found, and genetically identified samples and individuals in the Hudson and Mohawk River study areas in both sampling years (2013 and 2014).

	2013			2014		
	Hudson	Mohawk	Total	Hudson	Mohawk	Total
Sites surveyed	74	68	143	76	76	152
Days between surveys	24	26	—	23	25	—
*range*	17–33	20–33	—	13–34	16–37	—
Transect lengths	642 *m*	752 *m*	—	2,390 *m*	2,527 *m*	—
*range*	56–2,281	58–3,585	—	217–9,839	132–9,772	—
Sites with scat	50 (68%)	54 (79%)	104 (73%)	67 (88%)	62 (82%)	129 (85%)
Scat samples	279	780	1059	1218	1787	3005
Identified non-targets	23	23	46	72	17	89
Scat with individual ID	84	374	458	362	600	962
Individuals	30	85	115	78	123	201

‘Occupied‘ refers to the proportion of sites where scat was found without accounting for imperfect detection, i.e., *apparent* occupancy. ‘Days between surveys’ is the median number and ‘Transect lengths’ are mean lengths across all three surveys.

### Selecting the Detection Probability Model

**Figure 1:**
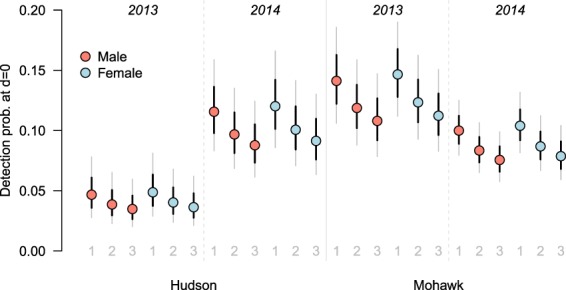
Variation in detectability. Baseline detection probability varied by session (unique river-year combinations: Hudson 2013, Hudson 2014, Mohawk 2013, Mohawk 2014), by visit (first, second and third visits), and by sex, although the latter received marginal support. Red and blue points are model averaged predictions of visit-specific baseline detection probability for each session and sex (red: males, blue: females). Bold black lines are unconditional standard errors of the predictions and the thin grey lines are the unconditional 95% confidence intervals.

Of the 32 candidate encounter models, those that relaxed the Euclidean assumption of circular and stationary space use (ecological distance models from here, see Methods), were systematically favored over the Euclidean distance models based on AIC (Supplementary Table S2, Fig. S7). Ecological distance models received a cumulative model weight of *ω*_+_ = 1.00 compared to *ω*_+_ = 0.00 for the Euclidean distance models. A model allowing the baseline detection probability (*p*_0_: probability of encounter when distance is 0) to vary by session was overwhelmingly preferred (*ω*_+_ = 0.91) to models with detection varying by river (*ω*_+_ = 0.02) or by year (*ω*_+_ = 0.04, Fig. 1). There was some support for sex difference in baseline detection probability (*ω*_+_ = 0.33), and complete support for sex-specific space use parameter, *σ* (*ω*_+_ = 1.00, Fig. 1, Supplementary Table S2).

In summary, ecological distance models that allowed for visit-specific detectability and sex-specific *σ* were preferred (Supplementary Table S2). Although there was support for models with and without sex differences (*ω*_+_ = 0.32 and 0.68, respectively), sex-specificity is biologically important and of interest. So as not to overly constrain the estimation of the detection function, we proceeded to analyze the 58 density models using both detection model structures: p(visit + session + sex) and p(visit + session), resulting in a total of 116 density models.

### Inference about Detection and Density

No single density model received overwhelming AIC support (Supplementary Table S1), so we used model averaging to account for model uncertainty^29^. The full model selection table is provided in the supplemental material (Supplementary Table S3) and, for clarity, we provide a reduced model selection table containing only models for which the 85% confidence intervals for all density covariates do not overlap zero^30^ (Table 2).

**Table 2:** Model selection table for the density models fitted in the second modeling step. For clarity, models containing variables with 85% confidence intervals included zero were removed^30^.

Density	Detection	${\boldsymbol{ {\mathcal L} }}$	np	AIC	ΔAIC	AIC_*ω*_	${\bf{A}}{\bf{I}}{{\bf{C}}}_{{\boldsymbol{\omega }}}^{{\boldsymbol{+}}}$
D(river)	p(session + visit)	3639.93	12	7303.85	0.00	0.63	0.63
D(river)	p(session + visit + sex)	3639.62	13	7305.23	1.38	0.31	0.94
D(river:d2stem)	p(session + visit)	3641.80	13	7309.60	5.75	0.04	0.97
D(river:d2stem)	p(session + visit + sex)	3641.51	14	7311.01	7.16	0.02	0.99
D(d2urban)	p(session + visit)	3644.97	12	7313.94	10.09	0.00	1.00
D(·)	p(session + visit)	3646.70	11	7315.40	11.55	0.00	1.00
D(d2urban)	p(session + visit + sex)	3644.73	13	7315.45	11.60	0.00	1.00
D(·)	p(session + visit + sex)	3646.46	12	7316.91	13.06	0.00	1.00

The model table shows the ‘Density’ and ‘Detection’ model structures, and the associated log-likelihood (${\mathcal L}$), number of parameters (np), AIC values, AIC differences (ΔAIC), model specific AIC model weights (AIC_*ω*_), and finally the cumulative AIC model weights for each model (${{\rm{AIC}}}_{\omega }^{+}$). All models had the same *σ* and model structure (σ ~ Sex). Models are ranked by AIC, lower AIC is more supported, and ΔAIC is the difference between each model and the model with the lowest AIC value.

The effects of ‘Visit’ and ‘Session’ on detection were present in every model and had full model support (i.e., *ω* + = 1, Table 3). The effect of sex, however, was present in only half of the models and had a relatively low RVI value (*ω* + = 0.33, Table 3). This suggests that, although there is non-zero support for differences in baseline detection probabilities between sexes, the differences are likely to be small or that we did not have sufficient power to detect the differences (Table 3, Supplementary Fig. S8).

**Table 3:** Model averaged coefficients for each parameter in the 116 density models.

Model	Covariate	Coefficient	*ω*+	$\hat{{\boldsymbol{\theta }}}$	se($\hat{{\boldsymbol{\theta }}}$)
Density	Intercept	*D*_Intercept_	1.00	−3.00	0.20
	River	*D*_Mohawk_	0.84	0.52	0.16
	Year	*D*_2014_	0.33	0.14	0.14
	Distance to Urban	*D*_d2urban_	0.32	−0.07	0.11
	Cover	*D*_cover_	0.36	−0.12	0.12
	Distance to Stem	*D*_d2stem_	0.31	0.01	0.03
	Session	*D*_session2_	0.12	0.07	0.32
		*D*_session3_	0.12	0.46	0.33
		*D*_session4_	0.12	0.62	0.32
	Interaction (d2stem-river)	*D*_d2stem:Hudson_	0.11	−0.01	0.05
		*D*_d2stem:Mohawk_	0.11	0.05	0.05
Detection	Intercept	*α*_0,Intercept_	1.00	−3.02	0.28
	Visit	*α*_0,visit2_	1.00	−0.20	0.09
		*α*_0,visit3_	1.00	−0.31	0.11
	Session	*α*_0,session2_	1.00	0.98	0.29
		*α*_0,session3_	1.00	1.21	0.29
		*α*_0,session4_	1.00	0.82	0.29
	Sex	*α*_0,male_	0.33	0.13	0.18
Sigma	Intercept	*α*_1,Intercept_	1.00	−1.57	0.06
	Sex	*α*_1,male_	1.00	0.50	0.06
ASU		*α*_2_	1.00	−1.23	0.11
Sex ratio		*ψ*	1.00	−1.13	0.18

The table shows the cumulative AIC weight: *ω*+, the model averaged estimate of each coefficient: $\hat{\theta }$, and the unconditional model averaged standard error: se($\hat{\theta }$). ASU is the asymmetric space use model parameter.

As expected, detectability was highest during the initial visit, the period with most accumulation time and before scat removal, lower during the second visit (*p*_0,visit2_ = −0.20 [95% CI −0.02–−0.38]), and lowest during the third visit (*p*_0,visit3_ = −0.31 [95% CI −0.09 −0.53]). In addition to visit-specific variation, detectability varied by session (i.e., by unique river-year combinations). Baseline detection probability was similar in the Hudson River in 2014 and the Mohawk River in 2013 and 2014, ranging from 0.08 to 0.15, but was markedly lower in the Hudson River in 2013, ranging from 0.03 to 0.05 (Supplementary Fig. S8). Estimates of *σ* were $\hat{\sigma }$_♂_ = 0.34 (95% CI: 0.29–0.39) and $\hat{\sigma }$_♀_ = 0.20 (95% CI: 0.18–0.23) for males and females respectively (Table 3), suggesting scatting area of male mink is larger than that of females. The estimate of the cost parameter determining how movement is influenced by riparian/non-riparian habitat was ${\hat{\alpha }}_{2}=-\,1.23$ [95% CI: −1.44–−1.02], which means that, for our binary cost surface (riparian = 0, non-riparian pixel = 1), the effective distance between two points on the landscape is closer when separated by non-riparian habitat (out-of-network) than by ‘within-network’ riparian habitat (see Discussion).

Variation in mink density was best predicted by ‘River’ (*ω* + = 0.85, Tables 2 and 3). The effect of ‘Year’, and ‘Session’ had much lower RVI values (*ω* + = 0.33 and 0.13, respectively) suggesting that the major source of variation at the study areas scale (i.e., the river systems) was between rivers, rather than between years or between river-year combinations. There was very little support for the within-river spatial predictors of mink density: ‘% cover’ (*ω* + = 0.36), ‘distance to urban’ (*ω* + = 0.32), ‘distance to stem’ (*ω* + = 0.31), and the ‘river-distance to stem’ interaction (*ω* + = 0.11).

**Figure 2:**
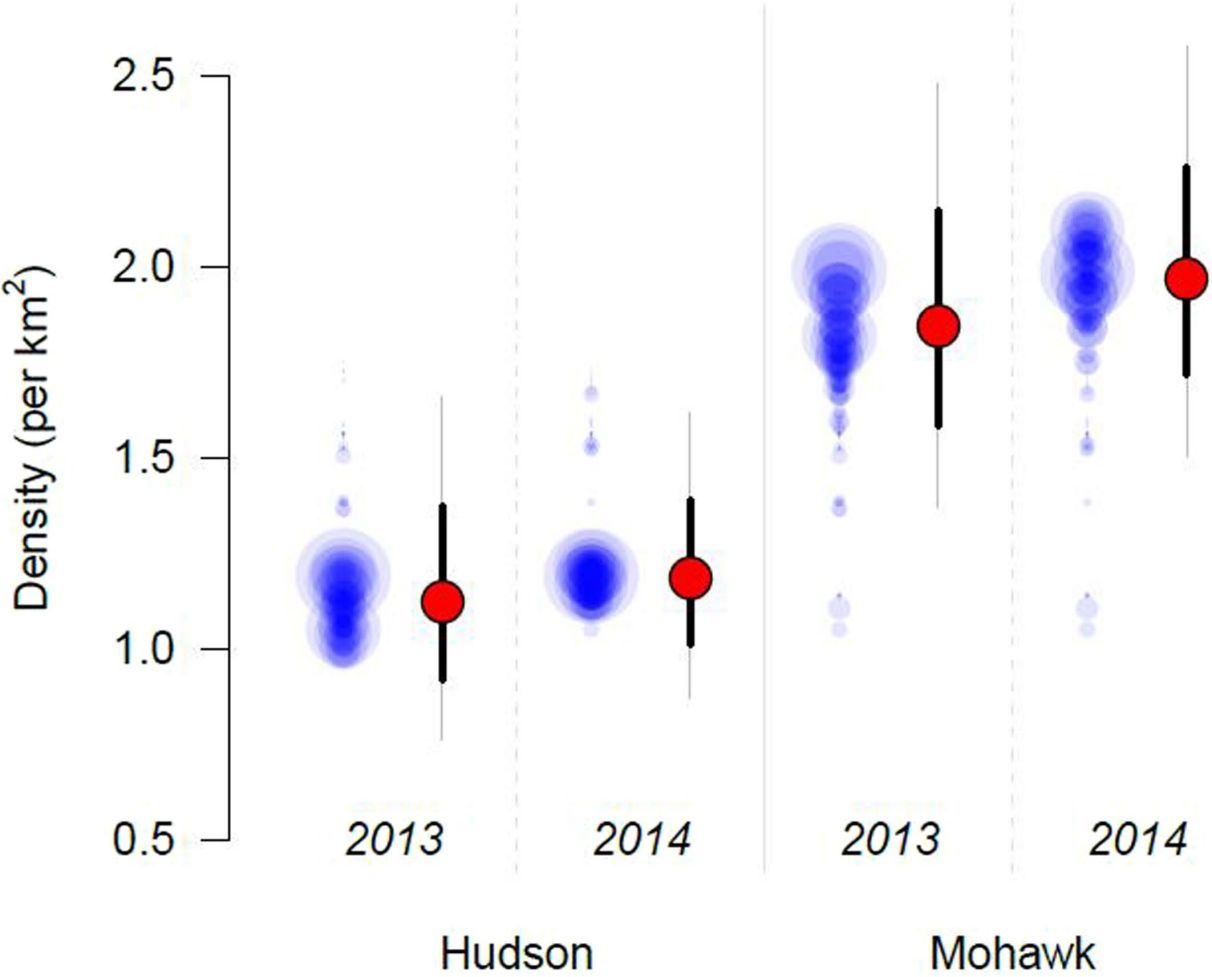
Variation in density. Density varied primarily by river (Hudson and Mohawk), although there was some support for year (2013, 2014) and session (unique river-year combinations: Hudson 2013, Hudson 2014, Mohawk 2013, Mohawk 2014) differences. Here we provide, on the left of each session, session-specific density for each of the 116 models as blue circles, where the size of the circles is proportional to the AIC model weight (${{\rm{AIC}}}_{\omega }^{+}$ in Table 2). On the right of each session, red points are model averaged predictions of session-specific density holding all continuous covariates, that had very little support and coefficient estimates not significantly different from 0, at their mean value (i.e., 0 because covariates were standardized). Bold black lines are unconditional standard errors of the predictions and the thin grey lines are the unconditional 95% confidence intervals.

There was strong support for models that allow variation by river only (‘River’: *ω* + = 0.84). In 2013, density was 1.64 times lower in the Hudson River than in the Mohawk: *D*_*M*,2013_ = 1.84 mink/*km*^2^ (95% CI: 1.37–2.48) and *D*_*H*,2013_ = 1.12 mink/*km*^2^ (95% CI: 0.76–1.66), respectively. In 2014, density was 1.67 times lower in the Hudson River than in the Mohawk: *D*_*M*,2014_ = 1.97 mink/*km*^2^ (95% CI: 1.50–2.57) and *D*_*H*,2014_ = 1.18 mink/*km*^2^ (95% CI: 0.87–1.62), respectively. In short, mink density differs between the Mohawk and Hudson Rivers, and density is lowest (~1.65 × lower) in the Hudson River (Fig. 2).

## Discussion

Our results provide compelling evidence that the density of American mink is significantly lower in the Hudson River than in the Mohawk River in both years (Fig. 2). Specifically, estimated mink density in the Hudson River was 1.64 and 1.67 times lower than the Mohawk River in 2013 and 2014, respectively. We found very little support for within-river spatial variation in density using vegetation cover, distance from urban areas and distance from the main stem as potential density predictors. Our objective was not to attribute variation explicitly to PCB contamination, but rather, investigate whether patterns of density were consistent with the hypothesis that extensive contamination has resulted in adverse effects on mink. So, whether the mechanism resulting in reduced densities in the Hudson are the result of direct impacts, e.g., on mink survival and/or fecundity, or indirect effects, e.g., on the riparian prey community, the fact remains that mink density varies substantially, and consistently, between two rivers that are similar in most ways other than the major difference in PCB contamination levels.

As expected, detectability decreased with successive surveys (Supplement Fig. S8). Scat encounter probability was highest in the occasion with the longest potential scat accumulation time, whereas after removal, and with on average 25 days between visits, scat detectability decreased. Detection was similar in all sampling sessions apart from in the Hudson River in 2013 (Supplement Fig. S8), the river-year sampling session with fewest scat samples collected, which may have been due to the extreme rainfall and flooding in 2013, although it is not clear whether this should have affected detectability in the Hudson more than in the Mohawk. Regardless of the fact that the source of this variability may not be clear, the session-by-visit structure of the detection model is flexible enough to account for these unknown sources of session-specific variation that density is estimated reliably with respect to the specification of the detection model.

Using the ecological distance formulation of the SCR model^31,32^ we were able to account for spatial asymmetry in expected encounter probabilities around an activity center that was explicitly related to the riparian landscape. Mink movement is typically assumed to have a higher degree of association with water networks or riparian corridors than areas away from water or non-riparian habitats^10,33,34^. Yet, contrary to expectation, we found that space use is *more* frequent between two points when separated by non-riparian habitat than the points separated by the same Euclidean distance along the water network (${\hat{\alpha }}_{2}=-\,1.23$, SE = 0.11). This result is in contrast to the positive resistance value reported in Fuller *et al*.^35^ which was based on a much smaller spatial extent than considered here (388 *km*^2^ vs. 1315 *km*^2^), and substantially fewer individuals detected at multiple traps (15 vs. 145). It is likely that these differences are scale-dependent and arise from the specific characteristics of the river network in the localized area considered in Fuller *et al*.^35^. In our study, the large spatial coverage and number of individuals is likely to remove such sensitivity to scale.

While counterintuitive, this makes sense biologically: although mink are unlikely to have symmetric (circular) home ranges, they are equally unlikely to have strictly linear home ranges. Instead, mink home ranges conceivably encompass multiple river sections that are in close proximity, and hence, when unconnected waterways separated by apparently unsuitable non-riparian habitat fall within an individual’s home range, movement between these locations may be more frequent than expected under the Euclidean assumption. In a sense, a stream acts as an attractant to a mink whose home range is located on a different but nearby stream. The estimated negative resistance parameter likely reflects that movement occurs more frequently across the non-riparian matrix to access resources within a territory that is not linear, as is often assumed, but rather that can encompass multiple disconnected waterways.

We note that it is possible that some within-river spatial variation in density exists that is related to prey availability (e.g.^36^). Measuring prey availability across the (large) spatial scales investigated here however, is prohibitively time intensive and was beyond the scope of this study. Two observations are noteworthy in this regard. First, the two river systems are very similar in terms of their hydrology and land use compositions (see methods), and second, the diets of mink in both rivers systems are similar both in terms of species composition and the proportional species make-up^37^.

The debate surrounding the use of focal species as indicators is largely focused on whether or not the chosen species adequately reflects the conditions of the system^3,4^. Thus, when adequately justified, certain species are viable indicators (e.g., diamondback terrapins^38^ clapper rails^39^). During the process of the Hudson River natural resource damage assessment, several independent conclusions provide such justification for using mink as an indicator species. First, PCB contamination levels greatly exceed that expected naturally^40^. Second, PCB burdens in aquatic species, including mink, are positively correlated with environmental levels^18,19^. Third, aquatic species make up the majority of mink diet^12,37^. And finally, experimental evidence shows that elevated PCB burdens cause direct physical damage, reduced survival, and reproductive impairment^23^. Thus, given the exposure history of individual mink to PCBs, and the fact that individual variation in vital rates has the potential to influence spatio-temporal population dynamics^22^, monitoring American mink is useful for understanding the potential impacts of PCB contamination on mink populations as well as on the aquatic ecosystem. In fact, American mink are an ideal candidate species for aquatic ecosystem assessment in general^8^.

Once an indicator species is selected, the challenge that remains is that the state of the focal species should be estimated precisely and over an area large enough to be considered representative of the ecosystem. We demonstrated how this can be achieved by combining three tools for monitoring rare, elusive or otherwise difficult to observe species: scat detection dogs to locate genetic material for species of interest, non-invasive genetic methods for identifying individuals, and spatial capture-recapture methods for inference from the resulting individual encounter history data. Doing so, we obtained large-scale spatially explicit estimates of American mink density in the Hudson River, a river system exposed to high levels of long-term PCB contamination, and the Mohawk River, a hydrologically independent river system. Estimated mink density in the Hudson River was lower than in the Mohawk River, consistent with the hypothesis that sustained contamination of aquatic ecosystems has had a negative impact on the species occupying those niches. The integration of spatial and genetic sampling and spatially explicit density estimation methods is now commonly adopted in ecological monitoring, and, as we have demonstrated, should be considered when conducting ecosystem assessments using indicator species.

## Methods

### The Hudson and Mohawk River Systems

This study focused on a 620 *km*^2^ area within a 5 *km* buffer centered on a 65.21 *km* section of the main stem of the Hudson River extending downstream of the known point source of the PCB contamination (Supplementary Fig. S1). As a reference, we selected a 695 *km*^2^ area within a 5 *km* buffer centered on a 71.53 *km* section of the main stem of the Mohawk River, a geographically close river system within the Hudson River drainage basin, but hydrologically independent from the influence of Hudson River plant PCB discharges (Supplementary Fig. S2). Both rivers have similar environmental characteristics, receive approximately 10 *cm* of rain per month during the May-July survey period, have a relative elevation change of approximately 35 *m*, are heavily managed water bodies with canal systems consisting of dams and locks, and have comparable land use and land cover (Supplementary Fig. S3). Recorded environmental PCB levels and mink PCB burdens are, however, markedly different between the two rivers: PCB levels are lower in the Mohawk River than in the Hudson River^18^. For example, it was estimated that the Hudson River contributed 90% of the PCB loadings south of where the rivers meet^41^. The Mohawk River thus provides a reasonable reference study area for comparing estimates of mink density.

### Sampling Locations and Timing

We identified all stream-road intersections in the study areas and then removed sites if 1) they consisted of more than 80% ‘developed’ within a 100 *m* radius based the 2006 National Land Cover Database^42^, 2) they did not have landowner access permission, or 3) on direct inspection, were either deemed unsafe for dogs and/or humans to survey, or had no surface water. This resulted in 506 and 567 potential sampling sites in the Hudson and Mohawk Rivers, respectively. Sites located within 1 *km* of each other were grouped into ‘clusters’ to reduce travel time between sites and to expose individual mink to detection at multiple locations. Sites with no neighboring sites within 1 *km*, were removed.

Using a swapping algorithm (Chapter 10 in^43^), and based on two survey teams sampling over 10-weeks in 2013, we selected 74 and 68 sites in the Hudson and Mohawk River study areas, respectively. In 2014, three survey teams sampled over the same 10-week period which allowed for additional sites to be surveyed and for transect lengths to extended (Table 1). Of the sites sampled in 2013, 69 and 65 sites were sampled in 2014 in the Hudson and Mohawk areas, respectively. All remaining accessible and suitable sites were then ranked based on the optimization procedure and, based on available effort, an additional 7 sites in the Hudson and 11 sites in the Mohawk were selected resulting in 76 sites in both rivers in 2014 (Table 1, Supplementary Figs S1 and S2). Sampling in both years was conducted between the end of the breeding season in April^44^, and the onset of juvenile dispersal in late July^10,45^. In both years, each site was visited three times between May 2 and July 15. Transect lengths, and the number of days between surveys are provided in Table 1.

### Scat Surveys and Individual Identification

Scat surveys were conducted by scat detection dogs, a handler and a data recorder surveying both banks of the river along the transects. Dogs wore GPS collars and searched off-leash and in-sight of the handler. When the scat was located by a dog, the exact location was recorded (see^35^). Fewer scat samples were found in 2013 compared to 2014 (1059 and 3005 respectively, Table 1), and in both years fewer scat samples were found in the Hudson River study area than in the Mohawk (Table 1). In the Hudson, 279 and 1218 scat samples were collected in 2013 and 2014 respectively and in the Mohawk, 780 and 1787 scat samples were collected in 2013 and 2014 respectively (Table 1). Scat samples were identified to individual level using 11 microsatellites using the methods described in detail in Fuller *et al*.^35^. In total there were 316 genetically identified mink (Table 1). Fewer mink were detected in the Hudson River study area in both years: 30 and 78 individuals in 2013 and 2014 respectively, compared to 85 and 123 in the Mohawk River (Table 1). Individual spatial encounter histories were constructed using the genetic identity, location, and sample occasion of each scat sample. Effective “traps” were created by converting GPS track lines from the dogs to 50 *m* × 50 *m* grid cells (Supplementary Fig. S4). This resulted in 817 and 1,857 trap locations in the Hudson in 2013 and 2014, respectively, and 805 and 1,854 in the Mohawk in the two years, respectively.

### Density Estimation

We used the standard SCR model assuming that *y*_*ijk*_, the observation of an individual (*i*) at a trap (*j*) during an occasion (*k*), are binomial random variables where the probability of *y* = 1 is a function of the distance, *d*(*x*_*j*_, *s*_*i*_), between an individual’s activity center, *s*_*i*_ and the location of the trap, *x*_*j*_:

1 $${\Pr }({y}_{ijk}=1)={p}_{0}\times \exp [-\,{\alpha }_{1,{\rm{sex}}}d{({x}_{j},{s}_{i})}^{2}],$$

where *p*_0_ is the baseline encounter probability, which could vary by individual, trap, and/or occasion attributes using a logit-link, logit(*p*0_*i*,*j*,*k*_) = *a*0_*i*,*j*,*k*_, and *α*_1_ = 1/(2*σ*^2^) controls the shape of the function. *d*(*x*_*j*_, *s*_*i*_) is the Euclidean distance between a trap and an individual activity center. Parameters *α*_0_ and *α*_1_ are parameters to be estimated.

To account for the unknown accumulation time in the first visit and the subsequent clearing of samples, we were interested in accounting for occasion-specific variation in detection probability. This is easily accommodated in SCR by modeling *p*_0_ as a function of a time-varying trap level covariate ‘visit’. Because accumulation times differed from visit one to visits two and three, a ‘visit’ effect was included in all models. We also tested for sex-specific variation in both the baseline detection probability and the spatial scale parameter, and allowed detection to vary by session, i.e., by year and river, by river only, and by year only. To accommodate the fact that sex determination was not made for all identified individuals, we applied a discrete 2-class mixture model to estimate the parameter *ψ*, the probability that an individual in the population is male^46^. All combinations of these effects produced a candidate set of 16 encounter models.

American mink are riparian habitat specialists and their movements are known to be closely associated with, but not necessarily *restricted* to, waterways^33,34^. This behavior potentially violates the Euclidean assumption that the detection kernel is symmetric and stationary (e.g.^32,35^). We adopted the ‘Ecological distance model’^31,32^ that allows explicit estimation of landscape resistance, and can therefore accommodate movement patterns that deviate from the circular assumption. For mink, a riparian specialist, we generated a 100 *m*^2^ resolution binary riparian cost surface where pixels that did not contain water were classified as non-riparian and had a value of 1, and pixels containing water were classified as riparian and were given a value of 0 (Supplementary Figs S4 and S5). This model represents an alternative, competing model to the Euclidean distance model with a single additional parameter, *α*_2_, determining the strength of the effect of landscape structure on space use. We fit an ecological distance analogue of each of the encounter models described above resulting in 32 detection models in total.

A point process model describes the distribution of individual activity centers across the landscape (i.e., density^43,47^). In addition to the standard uniform density model, spatial variation in density can be modeled using spatially varying covariates. For statistical convenience, the state space of the point process, *S*, is a discrete representation of the landscape described by pixel centroids. We use the standard log-linear model for *μ*(*s*, *β*), the per-pixel density, which can accommodate spatial covariates (*C*):

2 $$\log (\mu ({s}_{i},\beta ))={\beta }_{0}+\mathop{\sum }\limits_{v=1}^{V}{\beta }_{v}{C}_{i,v}.$$

The resolution of the state-space, and the scale at which all spatially explicit density covariates were computed, was 210 *m* × 210 *m* (Supplementary Figs S5 and S6). This resolution was considered fine enough to approximate a continuous landscape for mink while maintaining computational tractability and parameter insensitivity^43^. The state-space must be an area large enough to include all plausible activity centers of all detected individuals and, following recommendations by Sollmann *et al*.^48^, and based on the results from a pilot study^35^, we defined ${\mathscr{S}}$ as all pixels within a conservative 4.5 *km* of any trap. Because mink are semi-aquatic riparian specialists, mink activity centers, e.g., denning sites^49^, are likely to be located along the water network. For example, all denning sites of 13 telemetered individuals were within 50 *m* from water^50^, and in the Hudson River, almost all mink resting sites were located within 10 *m* of water^51^. As such, we created a biologically realistic state space that included only riparian areas. It is important to note that, while activity centers are restricted to the riparian corridors, space use is not restricted to the waterway but rather is affected by the waterway according to the ecological distance model.

We expected the distribution of mink to be influenced by proximity to urban areas^52^, and vegetative cover^53^. Distance to the nearest urban area, *D*_d2urban_, was calculated as the distance to the edge of the nearest area of high intensity urbanization, (≥10 *ha* contiguous 50–100% impervious surface based on the NLCD landcover data). Percent vegetation cover, *D*_cover_, was calculated as the percentage of overstory (deciduous, coniferous, and mixedwood trees >5 *m* tall) and/or understory (shrub/scrub <5 *m* tall) vegetation within each state-space pixel (Supplementary Figs S5 and S6).

We hypothesized that based on proximity to the pollutant source, densities in areas closer to the main stem of the Hudson River may be lower^18,19,23,24^. Therefore, we included a continuous ‘distance to main stem’ covariate, *D*_d2stem_, computed as the Euclidean distance from the center of each pixel to the main stem (Figs S4 and S5). We expected this gradient to differ between the contaminated Hudson River and the reference Mohawk River, so we included a river-distance interaction term that allows the effect of distance to vary between the two rivers.

Finally, our primary interest was to compare river-specific estimates of density while also accounting for potential variation in density across years. Therefore, we allowed density to vary by river but fixed across years, by year but fixed across rivers, and also by session (each unique river-year combination). All combinations of these density covariate models, including the uniform density model, resulted in a total of 58 candidate density models.

Testing all combinations of the detection and density models would result in a prohibitively large number of possible model combinations (≈2, 000), and instead, we adopted a two-step model fitting approach^54,55^. First, we fitted all 32 encounter models using a flexible session specific density model. Using AIC-based model selection^29^, we selected the most supported detection model structure(s) to proceed with the second modeling stage. Using the most supported detection model(s) we fitted and compared all 58 density models. Inference was based on this final model set which we ranked, weighted, and if necessary, averaged using AIC^29^. We calculated relative variable importance (RVI or *ω*+) for each covariate by summing the AIC model weights across each model in which the covariate was present. All models were fitted using the *R* package oSCR^56,57^, a package designed specifically for fitting multi-session sex-structured SCR models.

The data analysed and the code to conduct the analysis are available here^58^.

## Electronic supplementary material

Supplementary information
