# Author Correction: Large-scale variation in density of an aquatic ecosystem indicator species

**DOI:** 10.1038/s41598-019-53158-6

**Published:** 2019-11-06

**Authors:** Chris Sutherland, Angela K. Fuller, J. Andrew Royle, Matthew P. Hare, Sean Madden

**Affiliations:** 1 Department of Environmental Conservation, University of Massachusetts, Amherst, 01003 USA; 2 Department of Natural Resources, U.S. Geological Survey, New York Cooperative Fish and Wildlife Research Unit, Cornell University, Ithaca, 14853 USA; 3 U.S. Geological Survey, Patuxent Wildlife Research Center, Laurel, 12311 USA; 4 Department of Natural Resources, Cornell University, Ithaca, 14853 USA; 5 New York State Department of Environmental Conservation, Division of Fish and Wildlife, Albany, 12233 USA; 6 U.S. Geological Survey, New York Cooperative Fish and Wildlife Research Unit, Department of Natural Resources, Cornell University, Ithaca, 14853 USA

Correction to: *Scientific Reports* 10.1038/s41598-018-26847-x, published online 12 June 2018

Matthew P. Hare was omitted from the author list in the original version of this Article. This has been corrected in the PDF and HTML versions of the Article, and in the accompanying Supplementary Information file.

The Author Contributions section now reads:

C.S., A.K.F. and J.A.R. contributed equally to all parts of this manuscript. S.M. assisted in developing the study and contributed to parts of the writing of the manuscript. M.P.H. supervised and conducted the genetic analysis.

