# Supplementary material for: Large-scale variation in density of an aquatic ecosystem indicator species
Source: Sci Rep. 2018 Jun 12;8:8958. doi: 10.1038/s41598-018-26847-x (PMC5997698; doi:10.1038/s41598-018-26847-x)
Supplement: Supplementary file 1 — Supplementary information [file 41598_2018_26847_MOESM1_ESM.pdf]

Large-scale variation in density of an aquatic ecosystem indicator species

Chris Sutherland[1,*], Angela K Fuller[2,6], J. Andrew Royle[3], Matthew P Hare[4], and Sean Madden[5]

[1]University of Massachusetts, Department of Environmental Conservation, Amherst, 01003, USA
[2]Department of Natural Resources, U.S. Geological Survey, New York Cooperative Fish and Wildlife Research Unit, Cornell University, Ithaca, 14853, USA
[3]U.S. Geological Survey, Patuxent Wildlife Research Center, Laurel, 12311, USA
[4]Department of Natural Resources, Cornell University, Ithaca, 14853, USA
[5]New York State Department of Environmental Conservation, Division of Fish and Wildlife, Albany, 12233, USA
[6]U.S. Geological Survey, New York Cooperative Fish and Wildlife Research Unit, Department of Natural Resources, Cornell University, Ithaca, 14853, USA
[*]csutherland@umass.edu

ABSTRACT

This appendix contains the supplementary information cited in the main text.

Tables

Table S 1. Summary of the frequency of spatial recaptures. There were a total of 316 unique individuals caught, 145 of which were detected at more than one trap.

		Spatial encounters										
		1	2	3	4	5	6	7	8	9	10	>10
2013	Hudson	21	4	5	–	–	–	–	–	–	–	–
	Mohawk	43	12	10	4	3	3	–	2	–	–	–
2014	Hudson	47	15	3	6	4	5	1	–	1	1	2
	Mohawk	60	18	16	8	8	5	–	–	1	2	5

Table S 2. Model performance summaries for the 32 detection models fitted in the first of two modeling steps aimed at selecting the most supported parameterization of the detection model. The model table shows the 'Density', 'Detection', 'Sigma' and 'Cost' (asu: asymmetric space use) model structures, and the associated log-likelihood ($\mathscr{L}$), number of parameters (np), AIC values, AIC differences (ΔAIC), model specific AIC model weights (AIC_{ω}), and finally the cumulative AIC model weights for each model (AIC_{ω}^{+}). Models are ranked by AIC, lower AIC is more supported, and ΔAIC is the difference between each model and the model with the lowest AIC value.

Density	Detection	Sigma	Cost	$\mathscr{L}$	np	AIC	ΔAIC	AIC_{ω}	AIC_{ω}^{+}
D(session)	p(visit+session)	sig(sex)	asu(riparian)	3639.37	14	7306.74	0.00	0.62	0.62
D(session)	p(visit+session+sex)	sig(sex)	asu(riparian)	3639.13	15	7308.26	1.52	0.29	0.91
D(session)	p(visit+year)	sig(sex)	asu(riparian)	3644.52	12	7313.03	6.29	0.03	0.93
D(session)	p(visit)	sig(sex)	asu(riparian)	3645.79	11	7313.59	6.84	0.02	0.95
D(session)	p(visit+sex)	sig(sex)	asu(riparian)	3645.05	12	7314.09	7.35	0.02	0.97
D(session)	p(visit+year+sex)	sig(sex)	asu(riparian)	3644.13	13	7314.26	7.52	0.01	0.98
D(session)	p(visit+river)	sig(sex)	asu(riparian)	3645.43	12	7314.86	8.12	0.01	0.99
D(session)	p(visit+river+sex)	sig(sex)	asu(riparian)	3644.75	13	7315.51	8.77	0.01	1.00
D(session)	p(visit+session)	sig(sex)	asu(·)	3652.03	13	7330.06	23.31	0.00	1.00
D(session)	p(visit+session+sex)	sig(sex)	asu(·)	3651.80	14	7331.60	24.86	0.00	1.00
D(session)	p(visit+year)	sig(sex)	asu(·)	3658.26	11	7338.53	31.79	0.00	1.00
D(session)	p(visit+year+sex)	sig(sex)	asu(·)	3657.82	12	7339.65	32.91	0.00	1.00
D(session)	p(visit+river)	sig(sex)	asu(·)	3659.01	11	7340.03	33.29	0.00	1.00
D(session)	p(visit+river+sex)	sig(sex)	asu(·)	3658.20	12	7340.41	33.67	0.00	1.00
D(session)	p(visit+sex)	sig(sex)	asu(·)	3660.03	11	7342.07	35.33	0.00	1.00
D(session)	p(visit)	sig(sex)	asu(·)	3661.05	10	7342.09	35.35	0.00	1.00
D(session)	p(visit+session+sex)	sig(·)	asu(riparian)	3662.15	14	7352.30	45.56	0.00	1.00
D(session)	p(visit+session)	sig(·)	asu(riparian)	3665.24	13	7356.49	49.75	0.00	1.00
D(session)	p(visit+year+sex)	sig(·)	asu(riparian)	3667.24	12	7358.48	51.74	0.00	1.00
D(session)	p(visit+sex)	sig(·)	asu(riparian)	3668.75	11	7359.50	52.76	0.00	1.00
D(session)	p(visit+river+sex)	sig(·)	asu(riparian)	3668.69	12	7361.39	54.65	0.00	1.00
D(session)	p(visit+year)	sig(·)	asu(riparian)	3671.09	11	7364.18	57.44	0.00	1.00
D(session)	p(visit)	sig(·)	asu(riparian)	3674.33	10	7368.67	61.92	0.00	1.00
D(session)	p(visit+river)	sig(·)	asu(riparian)	3674.05	11	7370.09	63.35	0.00	1.00
D(session)	p(visit+session+sex)	sig(·)	asu(·)	3685.73	13	7397.47	90.73	0.00	1.00
D(session)	p(visit+year+sex)	sig(·)	asu(·)	3690.03	11	7402.06	95.32	0.00	1.00
D(session)	p(visit+session)	sig(·)	asu(·)	3689.55	12	7403.09	96.35	0.00	1.00
D(session)	p(visit+sex)	sig(·)	asu(·)	3693.22	10	7406.45	99.71	0.00	1.00
D(session)	p(visit+river+sex)	sig(·)	asu(·)	3693.15	11	7408.29	101.55	0.00	1.00
D(session)	p(visit+year)	sig(·)	asu(·)	3694.51	10	7409.03	102.29	0.00	1.00
D(session)	p(visit)	sig(·)	asu(·)	3698.70	9	7415.40	108.66	0.00	1.00
D(session)	p(visit+river)	sig(·)	asu(·)	3698.56	10	7417.11	110.37	0.00	1.00

Table S 3. Model performance summaries for the 116 density models fitted in the second modeling step. The model table shows the 'Density', 'Detection', 'Sigma' and Cost' model structures, and the associated log-likelihood ($\mathscr{L}$), number of parameters (np), AIC values, AIC differences (ΔAIC), model specific AIC model weights (AIC_{ω}), and finally the cumulative AIC model weights for each model (AIC_{ω}^{+}). Models are ranked by AIC, lower AIC is more supported, and ΔAIC is the difference between each model and the model with the lowest AIC value.

Rank	Density	Detection	$\mathscr{L}$	np	AIC	ΔAIC	AIC_{ω}	AIC_{ω}^{+}
1	D(river)	p(session+visit)	3639.93	12	7303.85	0.00	0.10	0.10
2	D(year+river)	p(session+visit)	3639.40	13	7304.80	0.95	0.06	0.16
3	D(river+cover)	p(session+visit)	3639.44	13	7304.87	1.02	0.06	0.22
4	D(river)	p(session+visit+sex)	3639.62	13	7305.23	1.38	0.05	0.27
5	D(river+d2urban)	p(session+visit)	3639.78	13	7305.57	1.72	0.04	0.31
6	D(river+d2stem)	p(session+visit)	3639.84	13	7305.68	1.83	0.04	0.35
7	D(year+river+cover)	p(session+visit)	3638.93	14	7305.86	2.01	0.04	0.38
8	D(river+cover)	p(session+visit+sex)	3639.11	14	7306.22	2.37	0.03	0.41
9	D(year+river)	p(session+visit+sex)	3639.16	14	7306.32	2.47	0.03	0.44
10	D(year+river+d2urban)	p(session+visit)	3639.27	14	7306.55	2.70	0.03	0.47
11	D(year+river+d2stem)	p(session+visit)	3639.30	14	7306.61	2.76	0.02	0.49
12	D(river+d2urban+cover)	p(session+visit)	3639.31	14	7306.63	2.78	0.02	0.52
13	D(river+d2stem+cover)	p(session+visit)	3639.36	14	7306.72	2.87	0.02	0.54
14	D(session)	p(session+visit)	3639.37	14	7306.74	2.89	0.02	0.56
15	D(river+d2urban)	p(session+visit+sex)	3639.48	14	7306.96	3.11	0.02	0.59
16	D(river+d2stem)	p(session+visit+sex)	3639.53	14	7307.06	3.21	0.02	0.61
17	D(river+d2urban+d2stem)	p(session+visit)	3639.63	14	7307.27	3.42	0.02	0.62
18	D(year+river+cover)	p(session+visit+sex)	3638.67	15	7307.35	3.50	0.02	0.64
19	D(river+d2stem+river:d2stem)	p(session+visit)	3639.76	14	7307.51	3.66	0.02	0.66
20	D(year+river+d2urban+cover)	p(session+visit)	3638.82	15	7307.64	3.79	0.01	0.67
21	D(year+river+d2stem+cover)	p(session+visit)	3638.84	15	7307.68	3.83	0.01	0.69
22	D(session+cover)	p(session+visit)	3638.90	15	7307.81	3.96	0.01	0.70
23	D(river+d2urban+cover)	p(session+visit+sex)	3638.99	15	7307.98	4.13	0.01	0.71
24	D(river+d2stem+cover)	p(session+visit+sex)	3639.03	15	7308.06	4.21	0.01	0.72
25	D(year+river+d2urban)	p(session+visit+sex)	3639.03	15	7308.06	4.21	0.01	0.74
26	D(year+river+d2stem)	p(session+visit+sex)	3639.06	15	7308.12	4.27	0.01	0.75
27	D(year+river+d2urban+d2stem)	p(session+visit)	3639.11	15	7308.21	4.36	0.01	0.76
28	D(session)	p(session+visit+sex)	3639.13	15	7308.26	4.41	0.01	0.77
29	D(river+d2urban+d2stem+cover)	p(session+visit)	3639.17	15	7308.35	4.50	0.01	0.78
30	D(year+river+river:d2stem)	p(session+visit)	3639.21	15	7308.42	4.57	0.01	0.79
31	D(session+d2urban)	p(session+visit)	3639.24	15	7308.48	4.63	0.01	0.80
32	D(session+d2stem)	p(session+visit)	3639.27	15	7308.54	4.69	0.01	0.81
33	D(river+d2urban+d2stem)	p(session+visit+sex)	3639.33	15	7308.65	4.80	0.01	0.82
34	D(river+d2stem+river:d2stem)	p(session+visit+sex)	3639.44	15	7308.88	5.03	0.01	0.83
35	D(river+d2urban+river:d2stem)	p(session+visit)	3639.47	15	7308.94	5.09	0.01	0.83
36	D(year+river+d2urban+cover)	p(session+visit+sex)	3638.56	16	7309.13	5.28	0.01	0.84

Rank	Density	Detection	$\mathscr{L}$	np	AIC	ΔAIC	AIC_{ω}	AIC_{ω}^{+}
37	D(year+river+d2stem+cover)	p(session+visit+sex)	3638.58	16	7309.16	5.31	0.01	0.85
38	D(session+cover)	p(session+visit+sex)	3638.65	16	7309.29	5.44	0.01	0.85
39	D(year+river+d2urban+d2stem+cover)	p(session+visit)	3638.67	16	7309.33	5.48	0.01	0.86
40	D(d2urban+d2stem+d2stem:river)	p(session+visit)	3640.69	14	7309.37	5.52	0.01	0.87
41	D(year+river+cover+river:d2stem)	p(session+visit)	3638.73	16	7309.47	5.62	0.01	0.87
42	D(session+d2urban+cover)	p(session+visit)	3638.79	16	7309.58	5.73	0.01	0.88
43	D(river:d2stem)	p(session+visit)	3641.80	13	7309.60	5.75	0.01	0.88
44	D(session+d2stem+cover)	p(session+visit)	3638.81	16	7309.62	5.77	0.01	0.89
45	D(river+d2urban+d2stem+cover)	p(session+visit+sex)	3638.85	16	7309.70	5.85	0.01	0.89
46	D(year+river+d2urban+d2stem)	p(session+visit+sex)	3638.87	16	7309.73	5.88	0.01	0.90
47	D(year+river+d2urban+river:d2stem)	p(session+visit)	3638.93	16	7309.87	6.02	0.00	0.90
48	D(year+river+river:d2stem)	p(session+visit+sex)	3638.97	16	7309.93	6.08	0.00	0.91
49	D(river+d2urban+cover+river:d2stem)	p(session+visit)	3638.99	16	7309.99	6.14	0.00	0.91
50	D(session+d2urban)	p(session+visit+sex)	3639.00	16	7310.00	6.15	0.00	0.92
51	D(session+d2stem)	p(session+visit+sex)	3639.03	16	7310.06	6.21	0.00	0.92
52	D(session+d2urban+d2stem)	p(session+visit)	3639.07	16	7310.14	6.29	0.00	0.93
53	D(river+d2urban+river:d2stem)	p(session+visit+sex)	3639.16	16	7310.32	6.47	0.00	0.93
54	D(session+d2stem+d2stem:river)	p(session+visit)	3639.18	16	7310.35	6.50	0.00	0.93
55	D(d2urban+d2stem+cover+d2stem:river)	p(session+visit)	3640.24	15	7310.47	6.62	0.00	0.94
56	D(d2stem+cover+d2stem:river)	p(session+visit)	3641.34	14	7310.67	6.82	0.00	0.94
57	D(year+d2urban+river:d2stem)	p(session+visit)	3640.36	15	7310.73	6.88	0.00	0.94
58	D(d2urban+d2stem+d2stem:river)	p(session+visit+sex)	3640.39	15	7310.78	6.93	0.00	0.95
59	D(year+river+d2urban+d2stem+cover)	p(session+visit+sex)	3638.41	17	7310.82	6.97	0.00	0.95
60	D(year+river+cover+river:d2stem)	p(session+visit+sex)	3638.47	17	7310.95	7.10	0.00	0.95
61	D(year+river+d2urban+cover+river:d2stem)	p(session+visit)	3638.48	17	7310.96	7.11	0.00	0.96
62	D(river:d2stem)	p(session+visit+sex)	3641.51	14	7311.01	7.16	0.00	0.96
63	D(session+d2urban+cover)	p(session+visit+sex)	3638.53	17	7311.07	7.22	0.00	0.96
64	D(year+d2stem+d2stem:river)	p(session+visit)	3641.54	14	7311.08	7.23	0.00	0.96
65	D(session+d2stem+cover)	p(session+visit+sex)	3638.55	17	7311.11	7.26	0.00	0.97
66	D(session+d2urban+d2stem+cover)	p(session+visit)	3638.63	17	7311.27	7.42	0.00	0.97
67	D(river+d2urban+cover+river:d2stem)	p(session+visit+sex)	3638.67	17	7311.34	7.49	0.00	0.97
68	D(year+river+d2urban+river:d2stem)	p(session+visit+sex)	3638.69	17	7311.38	7.53	0.00	0.97
69	D(session+cover+river:d2stem)	p(session+visit)	3638.70	17	7311.40	7.55	0.00	0.98
70	D(session+d2urban+d2stem)	p(session+visit+sex)	3638.83	17	7311.66	7.81	0.00	0.98
71	D(session+d2urban+river:d2stem)	p(session+visit)	3638.89	17	7311.78	7.93	0.00	0.98
72	D(d2urban+d2stem+cover+d2stem:river)	p(session+visit+sex)	3639.93	16	7311.85	8.00	0.00	0.98
73	D(year+d2urban+cover+river:d2stem)	p(session+visit)	3639.93	16	7311.86	8.01	0.00	0.98
74	D(session+d2stem+d2stem:river)	p(session+visit+sex)	3638.93	17	7311.86	8.01	0.00	0.98
75	D(d2stem+cover+d2stem:river)	p(session+visit+sex)	3641.02	15	7312.04	8.19	0.00	0.99
76	D(year+d2urban+river:d2stem)	p(session+visit+sex)	3640.12	16	7312.25	8.40	0.00	0.99
77	D(year+river+d2urban+cover+river:d2stem)	p(session+visit+sex)	3638.22	18	7312.44	8.59	0.00	0.99
78	D(year+d2stem+d2stem:river)	p(session+visit+sex)	3641.29	15	7312.58	8.73	0.00	0.99
79	D(session+d2urban+d2stem+cover)	p(session+visit+sex)	3638.38	18	7312.76	8.91	0.00	0.99
80	D(session+d2urban+cover+river:d2stem)	p(session+visit)	3638.44	18	7312.88	9.03	0.00	0.99
81	D(session+cover+river:d2stem)	p(session+visit+sex)	3638.44	18	7312.89	9.04	0.00	0.99
82	D(session+d2urban+river:d2stem)	p(session+visit+sex)	3638.65	18	7313.30	9.45	0.00	0.99
83	D(year+d2urban+cover+river:d2stem)	p(session+visit+sex)	3639.68	17	7313.35	9.50	0.00	1.00
84	D(d2urban)	p(session+visit)	3644.97	12	7313.94	10.09	0.00	1.00
85	D(session+d2urban+cover+river:d2stem)	p(session+visit+sex)	3638.19	19	7314.37	10.52	0.00	1.00

Rank	Density	Detection	$\mathscr{L}$	np	AIC	ΔAIC	AIC_{ω}	AIC_{ω}^{+}
86	D(d2urban+cover)	p(session+visit)	3644.61	13	7315.22	11.37	0.00	1.00
87	D(·)	p(session+visit)	3646.70	11	7315.40	11.55	0.00	1.00
88	D(d2urban)	p(session+visit+sex)	3644.73	13	7315.45	11.60	0.00	1.00
89	D(d2urban+d2stem)	p(session+visit)	3644.93	13	7315.86	12.01	0.00	1.00
90	D(year+d2urban)	p(session+visit)	3644.96	13	7315.92	12.07	0.00	1.00
91	D(cover)	p(session+visit)	3646.33	12	7316.65	12.80	0.00	1.00
92	D(d2urban+cover)	p(session+visit+sex)	3644.35	14	7316.70	12.85	0.00	1.00
93	D(·)	p(session+visit+sex)	3646.46	12	7316.91	13.06	0.00	1.00
94	D(d2urban+d2stem+cover)	p(session+visit)	3644.57	14	7317.15	13.30	0.00	1.00
95	D(year+d2urban+cover)	p(session+visit)	3644.60	14	7317.20	13.35	0.00	1.00
96	D(d2urban+d2stem)	p(session+visit+sex)	3644.69	14	7317.37	13.52	0.00	1.00
97	D(d2stem)	p(session+visit)	3646.69	12	7317.37	13.52	0.00	1.00
98	D(year)	p(session+visit)	3646.70	12	7317.40	13.55	0.00	1.00
99	D(year+d2urban)	p(session+visit+sex)	3644.72	14	7317.45	13.60	0.00	1.00
100	D(year+d2urban+d2stem)	p(session+visit)	3644.92	14	7317.84	13.99	0.00	1.00
101	D(cover)	p(session+visit+sex)	3646.07	13	7318.14	14.29	0.00	1.00
102	D(d2stem+cover)	p(session+visit)	3646.31	13	7318.62	14.77	0.00	1.00
103	D(d2urban+d2stem+cover)	p(session+visit+sex)	3644.32	15	7318.63	14.78	0.00	1.00
104	D(year+cover)	p(session+visit)	3646.32	13	7318.65	14.80	0.00	1.00
105	D(year+d2urban+cover)	p(session+visit+sex)	3644.35	15	7318.70	14.85	0.00	1.00
106	D(d2stem)	p(session+visit+sex)	3646.44	13	7318.88	15.03	0.00	1.00
107	D(year)	p(session+visit+sex)	3646.45	13	7318.90	15.05	0.00	1.00
108	D(year+d2urban+d2stem+cover)	p(session+visit)	3644.57	15	7319.13	15.28	0.00	1.00
109	D(year+d2urban+d2stem)	p(session+visit+sex)	3644.68	15	7319.37	15.52	0.00	1.00
110	D(year+d2stem)	p(session+visit)	3646.69	13	7319.37	15.52	0.00	1.00
111	D(d2stem+cover)	p(session+visit+sex)	3646.05	14	7320.10	16.25	0.00	1.00
112	D(year+cover)	p(session+visit+sex)	3646.06	14	7320.12	16.27	0.00	1.00
113	D(year+d2stem+cover)	p(session+visit)	3646.31	14	7320.61	16.76	0.00	1.00
114	D(year+d2urban+d2stem+cover)	p(session+visit+sex)	3644.32	16	7320.63	16.78	0.00	1.00
115	D(year+d2stem)	p(session+visit+sex)	3646.44	14	7320.87	17.02	0.00	1.00
116	D(year+d2stem+cover)	p(session+visit+sex)	3646.04	15	7322.08	18.23	0.00	1.00

Figures

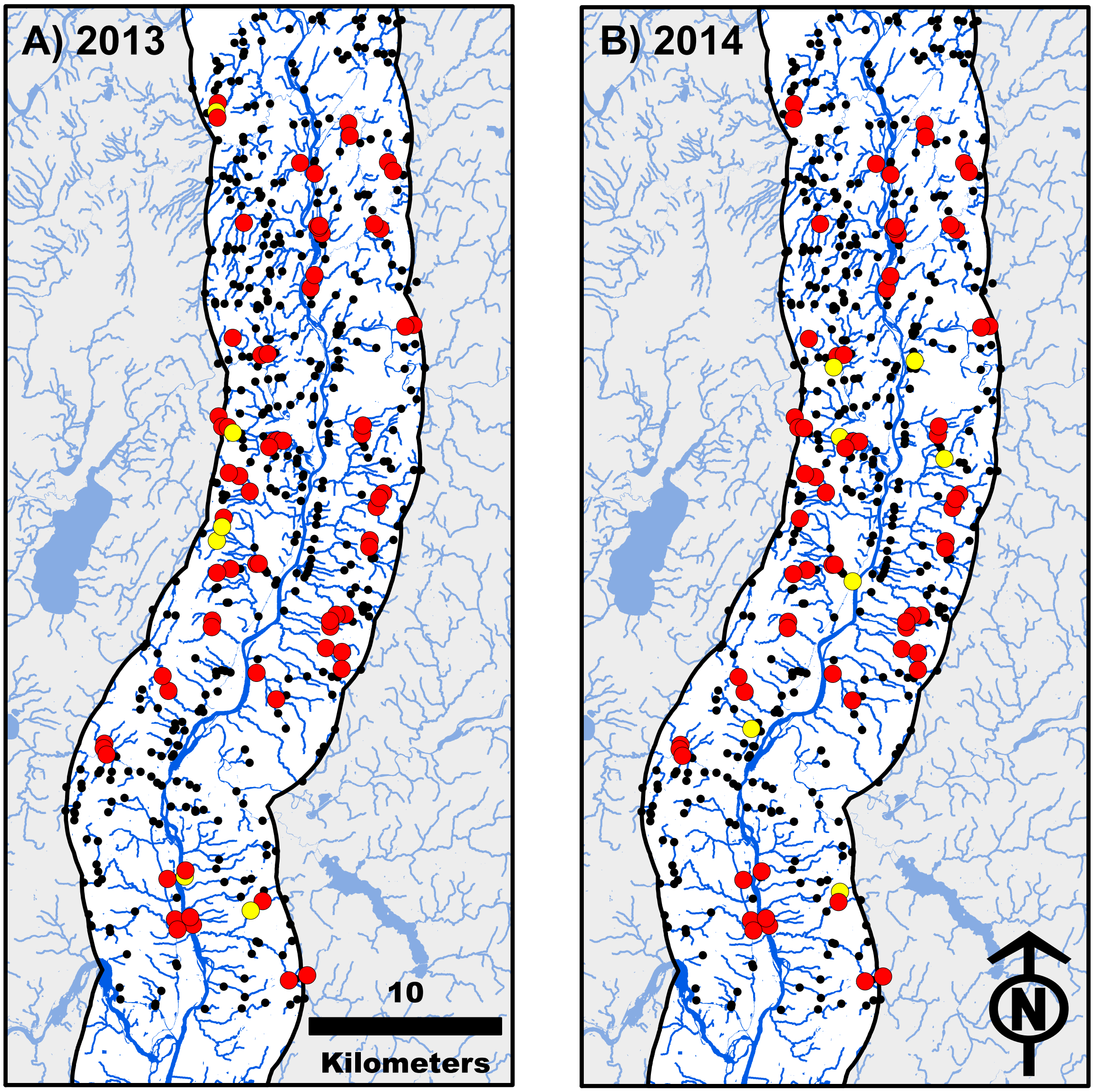

Figure S 1. The spatial distribution of sites within the Hudson River study area. Larger points denote sites that were selected for sampling and where scat transects were conducted (red: sampled in both years, yellow: sampled only in year shown). The smaller black points denote all other suitable sites not selected for sampling. Sites were selected based on minimizing the criterion $Q_{\bar{p}}$ using an optimization algorithm described above. Water bodies are represented by blue polygons, the main stem is represented by the thick blue line dissecting the buffer region, thin blue lines are tributaries, thin grey lines show the road network, and the grey shaded areas represent areas that lie beyond the 5 *km* buffer which is represented by the thick black line.

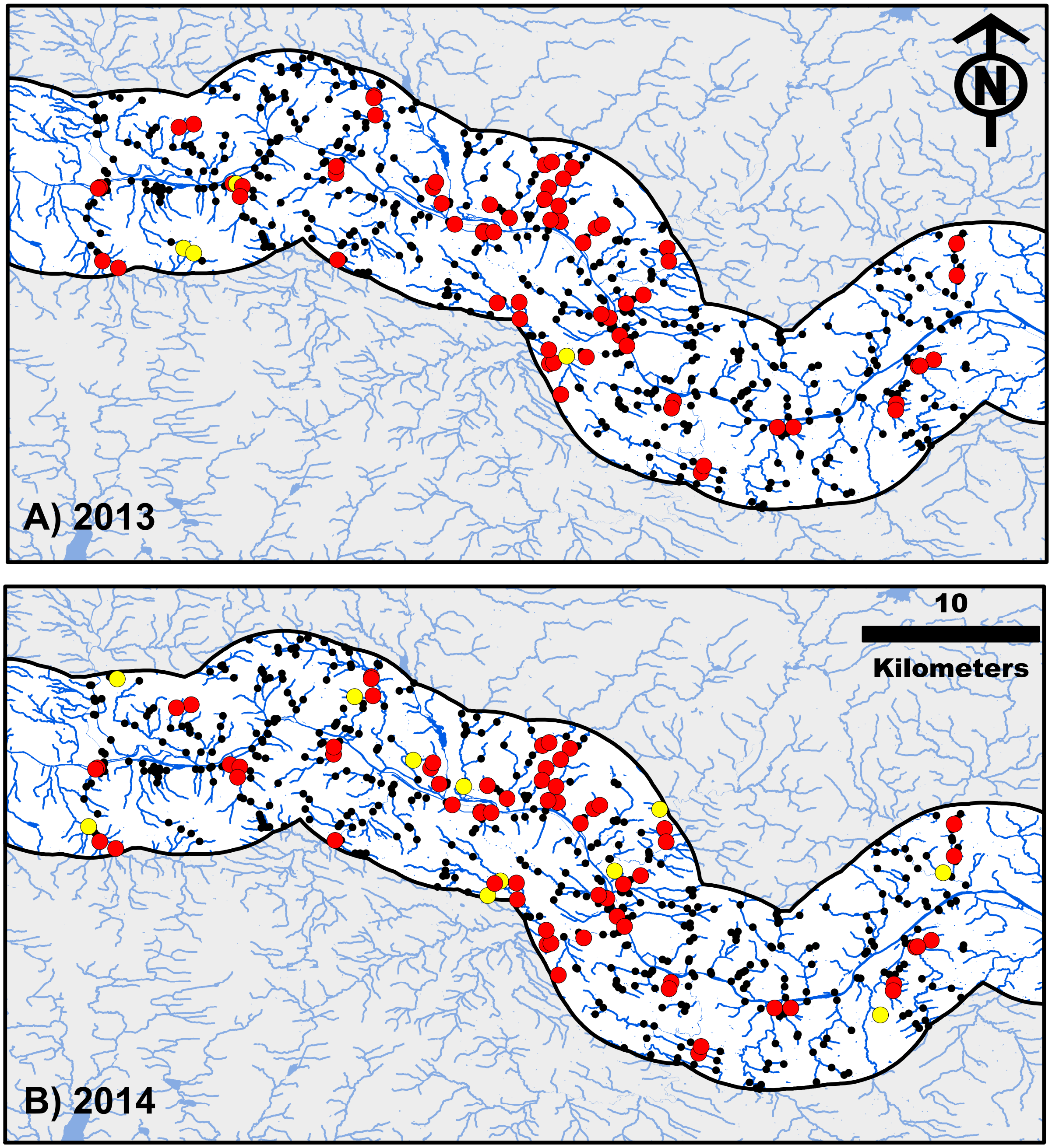

Figure S 2. The spatial distribution of sites within the Mohawk River study area. Larger points denote sites that were selected for sampling and where scat transects were conducted (red: sampled in both years, yellow: sampled only in year shown). The smaller black points denote all other suitable sites not selected for sampling. Sites were selected based on minimizing the criterion $Q_{\bar{p}}$ using an optimization algorithm. Water bodies are represented by blue polygons, the main stem is represented by the thick blue line dissecting the buffer region, thin blue lines are tributaries, thin grey lines show the road network, and the grey shaded areas represent areas that lie beyond the 5 *km* buffer which is represented by the thick black line.

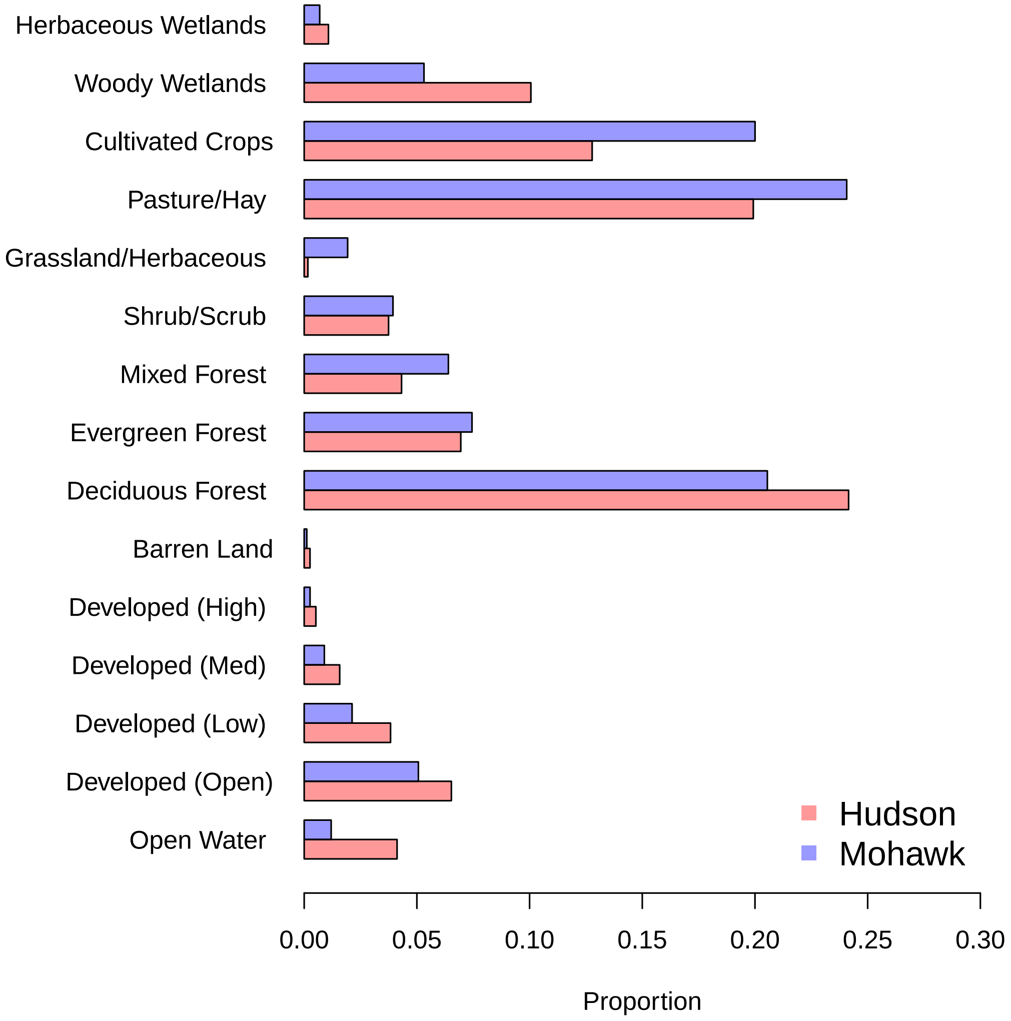

Figure S 3. A comparison of the proportional composition of landcover types in the Hudson and Mohawk rivers. Proportions are calculated by dividing the number of 30 m × 30 m pixels of each landcover class by the total number of 30 m × 30 m pixels in the study area.

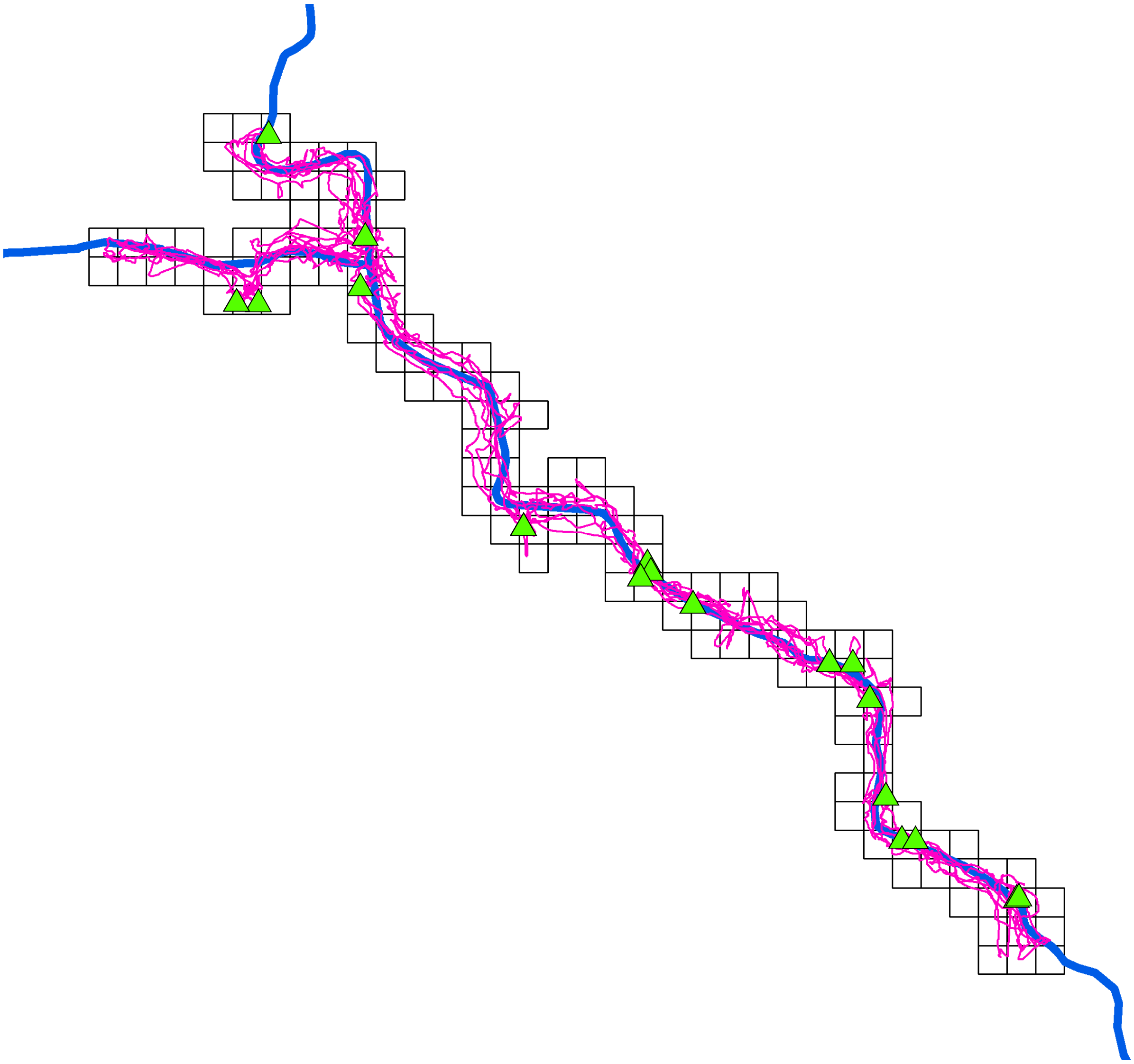

Figure S 4. Effective traps were constructed by identifying every 50 $m \times 50$ m grid cell that was traversed during a transect along a stream. This figure is a schematic of the construction of traps for a single transect. Pink lines are scat detection dog GPS track logs (the transect line), the blue line is the stream along which the transect was conducted, the black grids are 50 m $\times$ 50 m cells intersected by the transect line, i.e., the effective traps. The green triangles are locations of scat, and for the generation of spatial encounter histories, scat are associated with the trap within which they were found.

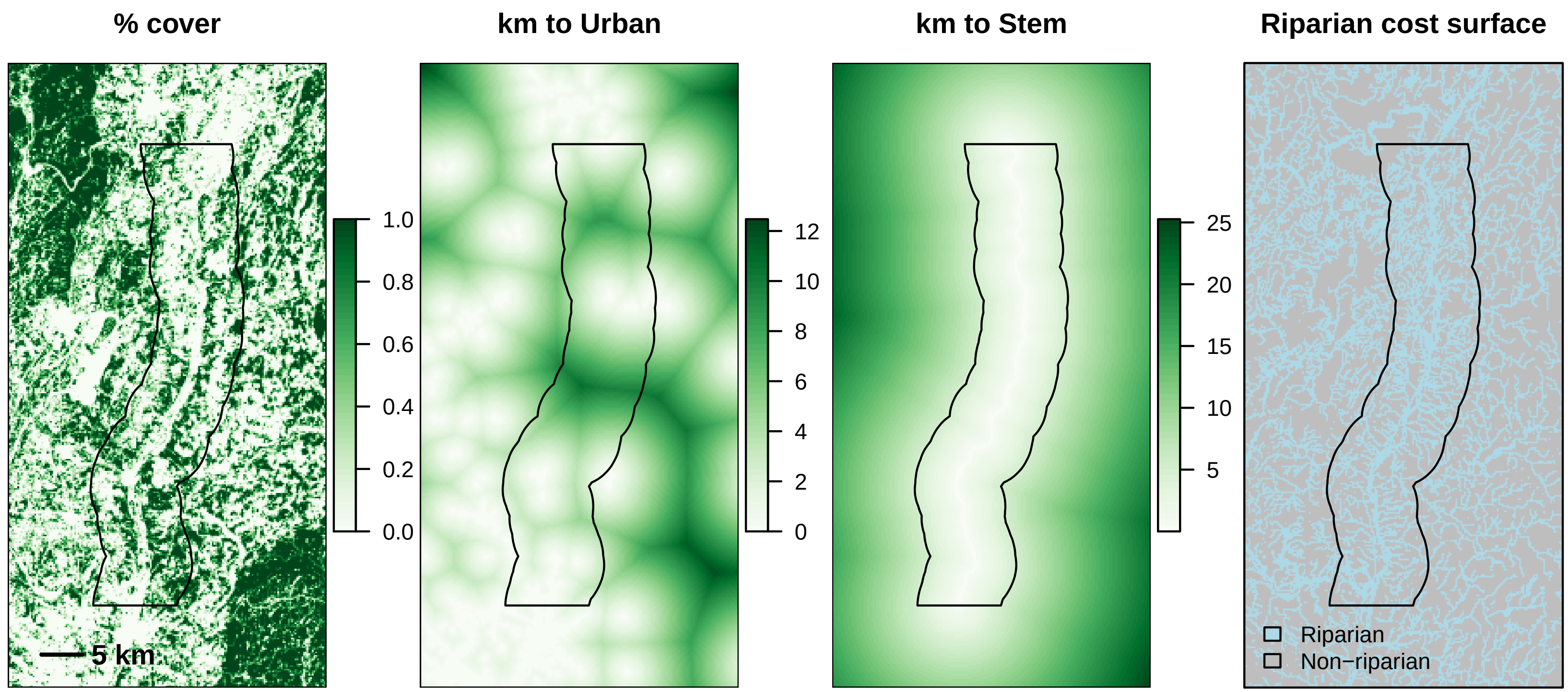

Figure S 5. The Hudson River habitat covariate surfaces used in the models. The covariates ‘%cover’ (0 = 0%, 1 = 100%), ‘km to Urban’ (in *km*), and ‘km to Stem‘ (in *km*) were used to account for spatial variation in density. The ‘Riparian’ surface (0 = Riparian, 1 = Non-riparian) was used to account for asymmetry in space use related to the river network.

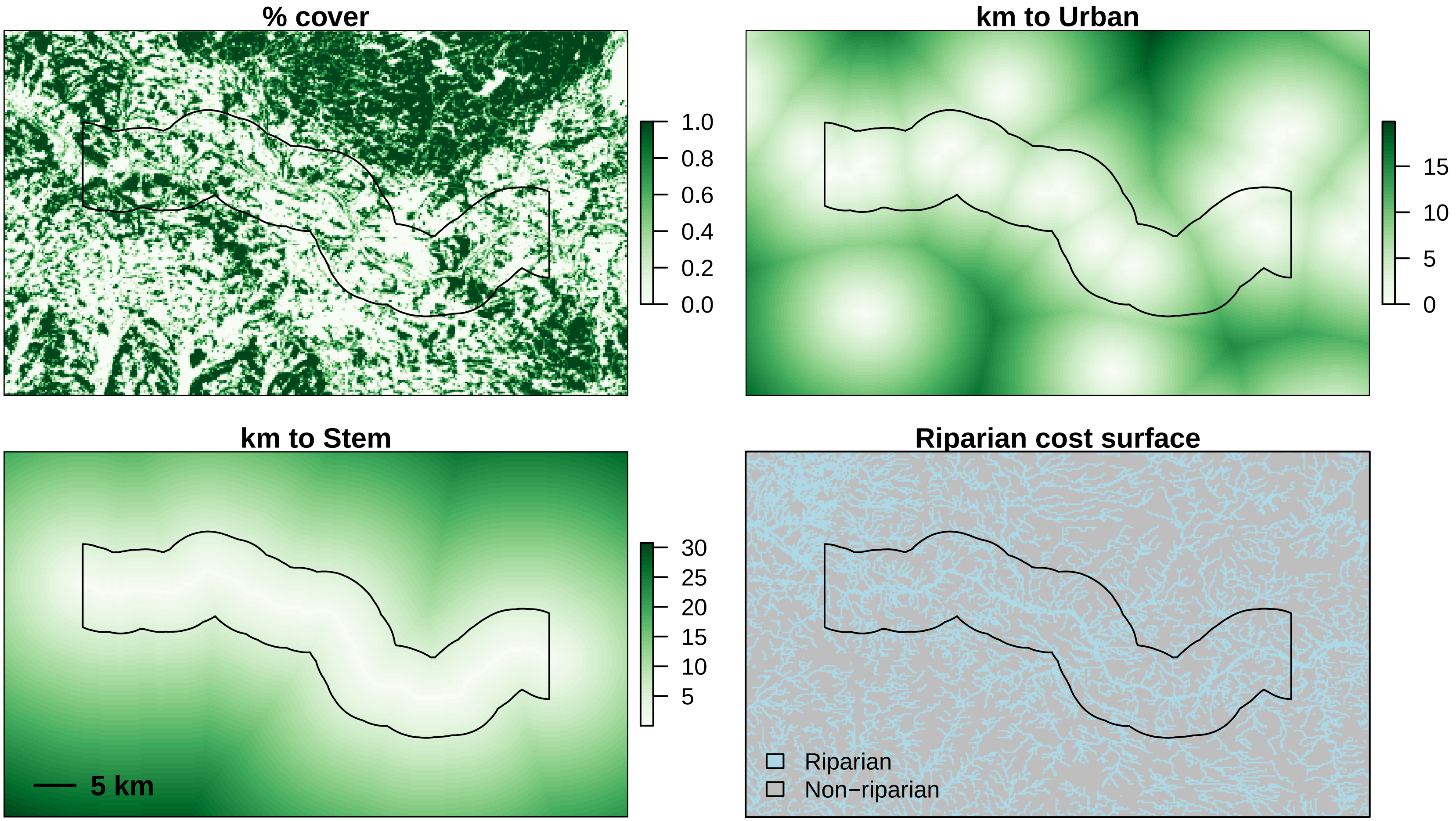

Figure S 6. The Mohawk River habitat covariate surfaces used in the models. The covariates '%cover' (0 = 0%, 1 = 100%), 'km to Urban' (in *km*), and 'km to Stem' (in *km*) were used to account for spatial variation in density. The 'Riparian' surface (0 = Riparian, 1 = Non-riparian) was used to account for asymmetry in space use related to the river network.

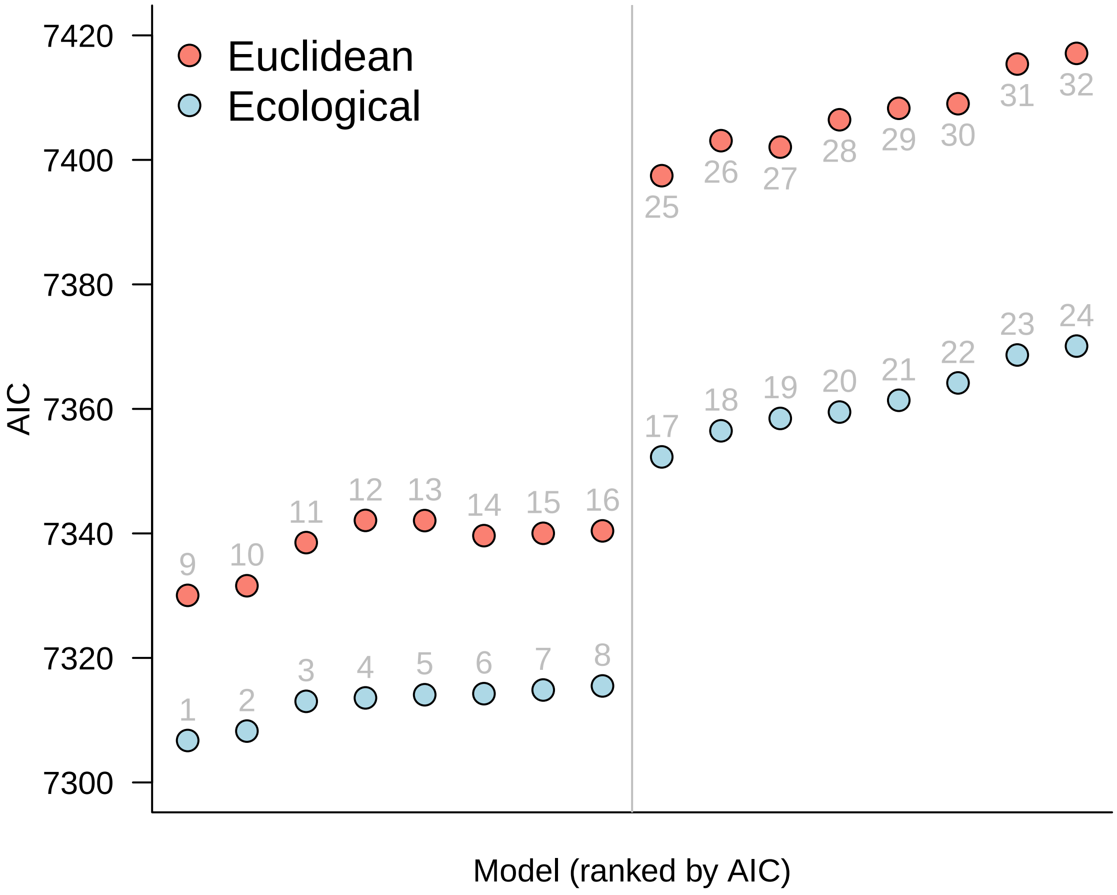

Figure S 7. Comparison of AIC values between the Euclidean distance model (light red circles) and ecological distance model (light blue circles). Here the asymmetric space use (ASU) models are ranked by AIC from lowest to highest and the Euclidean distance models are ranked in the same order as their ASU counterparts. For example, model 1 and model 9 are equivalent in all respects apart from the distance function; model 1 is the ASU model and model 9 is the Euclidean model. Models to the left of the grey line are models where σ is sex specific (σ_{sex}) and models to the right of the grey line are models with a constant σ ($\sigma_{.}$).
